# Radiosensitization of head and neck cancer by targeting the epidermal growth factor receptor

**DOI:** 10.4103/0971-5851.64252

**Published:** 2009

**Authors:** Arun Kumar Goel, Dinesh Singh

**Affiliations:** Departments of Surgical Oncology, Galaxy Cancer Institute, Pushpanjali Crosslay Hospital, Vaishali, Ghaziabad, India E-mail: arun.goel07@gmail.com; 1 Departments of Radiation Oncology, Galaxy Cancer Institute, Pushpanjali Crosslay Hospital, Vaishali, Ghaziabad, India

Radiotherapy plus cetuximab for locoregionally advanced head and neck cancer: 5-year survival data from a phase 3 randomized trial, and relation between cetuximab-induced rash and survival. Bonner JA, Harari PM, Giralt J, *et al*. Lancet Oncol 2010; 11: 21–28

This is the updated report on the outcome of the locally advanced head and neck squamous cell cancer, treated with external beam radiation with or without concomitant administration of a monoclonal antibody against the epidermal growth factor receptor (cetuximab). The first report[1] was published in the New England Journal of Medicine, in 2006.

The trial enrolled patients from April 1999 to March 2002. In this period, 424 patients with locally advanced (non-metastatic) head and neck cancers of the oropharynx, hypopharynx, and larynx (TNM stage groups III and IV) were randomly assigned to two treatment arms. The control arm consisted of the external beam radiation therapy (EBRT), without any concomitant radiosensitization. The experimental arm used concomitant administration of cetuximab along with EBRT. The trial was multicentric (73 centers in USA and 14 centers outside USA) and the patients were randomly assigned with the stratification of performance status, nodal involvement, T stage, and radiation fractionation regimen. The trial was unblinded as the administration of cetuximab was associated with an acneform skin rash in many patients that was not seen in the control arm.

Three different radiation protocols were allowed: once daily fractionation with 2 Gy fractions to a total dose of 70 Gy to gross disease; in the hyperfractionation scheme, twice daily fractions of 1.2 Gy each (with spacing of more than six hours between the two fractions) to a total dose of 72.0 to 76.8 Gy; and a third scheme with a concomitant boost (1.8 Gy per day for 30 fractions along with a 1.5 Gy second fraction during the last 12 days of treatment to a total dose of 72 Gy).

In the experimental arm; cetuximab was administered starting with a loading dose of 400 mg/m^2^ one week before start of radiation therapy (RT) and 250 mg/m^2^ infusion given every week for seven weeks. The patients were assessed regularly starting four weeks after completion of RT with four monthly assessments in the first two years and six monthly assessments subsequently.

In both arms, nearly 80% of the patients were male; nearly 80% were having nodal involvement; 72% patients were T1 to T3 in both arms, and 28% were T4. Nearly 60% of the patients had oropharyngeal primary (the percentage was slightly higher in RT alone arm). About 25% patients had AJCC stage III and the remaining had American Joint Committee on Cancer (AJCC) stage IV in both arms. Fifty six percent of the patients received RT in both arms, with a concomitant boost.

The median overall survival was 29.3 months in the control arm and 49.0 months in the experimental arm (RT and cetuximab). Five-year overall survival was 36.4% in the control arm and 45.6% in the experimental arm (hazard ratio 0.73 with *P* value 0.018). Subgroup analysis was carried out, showing a benefit in all the subgroups. An attempt was made to look for factors correlating with a higher or lower benefit from the addition of cetuximab. Patients with oropharyngeal cancers, early T stage, concomitant boost RT, higher N stage, higher performance status, male sex, and younger age were associated with a higher benefit from cetuximab.

Another observation was that patients who had a more prominent rash had a longer overall survival compared to those with milder rash. The median survival was 68.8 months versus 25.6 months in the two groups of patients (hazard ratio 0.49; *P* value 0.002).

Based on these results, the authors conclude that cetuximab provides a long-term and clinically significant survival advantage when administered concomitantly with radiation in locally advanced squamous cancers of the head and neck. These findings support the consideration of radiation with cetuximab as a viable option in the management of locally advanced cancers of the oropharynx, hypopharynx, and larynx.

## COMMENTS

In the earlier publication based on the same study, there was significant improvement in locoregional control with addition of cetuximab to RT[1]. The median duration of the locoregional control was 24.4 months with combined treatment, compared to 14.9 months with RT alone. Locoregional control with RT alone was 55, 41, and 34% at one, two, and three years. Locoregional control with combined therapy was 63, 50, and 47% at one, two. and three years. There was a 32% reduction in the risk of locoregional progression with addition of cetuximab. Survival at two years and three years was 55 and 45% with RT alone and 62 and 55% with RT plus cetuximab. Distant metastases were noticed in 17 and 16% patients of RT alone and combined therapy arms at two years follow up. Second primary cancers were noted in 5% of RT arm and 8% of combined therapy arm at two years follow up.

Overall, the two publications document the significant benefit that can be achieved with addition of cetuximab to radiation therapy, in terms of locoregional control, progression-free survival, and overall survival. Furthermore, the survival benefit becomes noticeable in the second year of follow up and persists without any decrease up to at least five years of follow up. It is quite reasonable to assume that this survival benefit is a sustained benefit, as the incidence of locoregional recurrences after five years of follow up is negligible in head and neck cancers. More important in this group of patients is the relatively high incidence of second malignancies. These facts are supported by the relatively low rate of distant metastases and the significant incidence of second malignancies at two years of follow up.

There are some important considerations yet. One is that the control arm in this study was radiation therapy alone. Currently, a majority of patients with locally advanced squamous cancers of the oropharynx, hypopharynx, and larynx are treated with concomitant radiotherapy and chemotherapy. Radiotherapy alone is used only in patients with poor performance status or other factors, such as, old age, significant co-existing medical problems, palliative therapy, and so on. Concomitant chemoradiotherapy has been shown to confer a significant advantage in locoregional control as well as survival and it has been confirmed in the most recent meta-analysis reported in 2008.[2] The meta-analysis has been referred to by the authors as well. It included 87 trials and 16485 patients. Based on 50 trials that used concomitant chemoradiotherapy, there was a 6.5% gain in overall survival at five years for patients treated with combined modality treatment.

The authors of the current study also raise the issue of significant toxicity seen with concurrent chemoradiotherapy. The current study showed that radiation-related toxicity in the group of patients receiving cetuximab was not higher than in the group treated with radiation alone. It would be reasonable to propose that a three-arm trial that compares concurrent chemoradiotherapy, RT with cetuximab and RT with chemotherapy and cetuximab could be useful in answering various questions that exist currently as well as show if combining the two concurrent strategies (cytotoxic chemotherapy and targeted therapy) would provide additional benefit to these patients.

Although cetuximab may find easy acceptance in USA and Europe, in resource-poor countries of Asia and Africa, it is important to consider the cost-benefit equation also in relation to concurrent chemotherapy and concurrent cetuximab. The most popular regimen for concurrent chemotherapy today is weekly administration of cisplatin. This regimen is highly effective and very cheap. In contrast, cetuximab will cost more than six lakh rupees for the total course of treatment. Unless the efficacy and toxicity profile of a cetuximab-based combined therapy shows significant benefit in direct comparison to Cisplatin-based combined therapy, it would be difficult to recommend cetuximab usage as concurrent therapy in Indian patients.

It would be important to explore if there are any patient groups that do not benefit or if there are any subgroups that show a higher-than-average benefit (from addition of cetuximab). The epidermal growth factor receptor (EGFR) expression was studied by the authors of the current study in nearly 80% of the patients and almost all the patients had documented expression of EGFR in the tumor cells. The proportion of cells expressing EGFR was more than 50% in nearly half of the patients studied. It might be necessary to look for other biological markers in the EGFR pathway to see if they showed a correlation with the benefit derived from concomitant usage of cetuximab.

There are many targeted agents against EGFR and it will be interesting to speculate about the relative efficacy of different agents in the setting of radiosensitization. Nimotuzumab is another monoclonal antibody targeted against EGFR. Early data from phase IIb studies in India have been reported recently and show benefit from addition of nimotuzumab to radiation alone or to radiation with concurrent chemotherapy.[3] Furthermore, phase III studies are necessary to document these benefits in a larger group. It may be preferable to have concurrent chemoradiation as the control arm and addition of nimotuzumab must be the study variable.
